# Vibration-mediated Kondo transport in molecular junctions: conductance evolution during mechanical stretching

**DOI:** 10.3762/bjnano.6.249

**Published:** 2015-12-17

**Authors:** David Rakhmilevitch, Oren Tal

**Affiliations:** 1 Department of Chemical Physics, Weizmann Institute of Science, Rehovot, Israel

**Keywords:** correlated systems, electron–phonon interactions, Kondo effect, molecular junctions, vibrations

## Abstract

The vibration-mediated Kondo effect attracted considerable theoretical interest during the last decade. However, due to lack of extensive experimental demonstrations, the fine details of the phenomenon were not addressed. Here, we analyze the evolution of vibration-mediated Kondo effect in molecular junctions during mechanical stretching. The described analysis reveals the different contributions of Kondo and inelastic transport.

## Introduction

**Figure 1:**
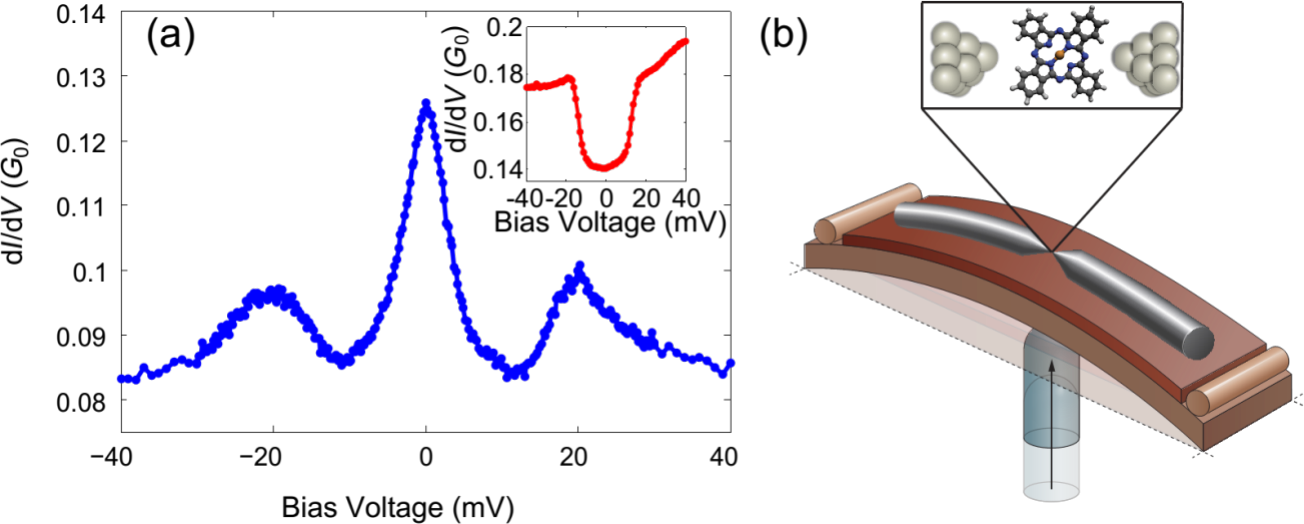
(a) Differential conductance vs applied bias voltage (d*I/*d*V* vs *V*) measured for a silver/copper–phthalocyanine (Ag/CuPc) molecular junction. The spectrum exhibits a zero-bias Kondo peak accompanied by vibration-mediated Kondo side peaks [23]. Inset: A vibration-induced differential conductance step occurring in the absence of a zero-bias Kondo effect. (b) Schematic representation of a mechanically controllable break junction device.

Molecular junctions are an attractive test-bed for electronic effects such as Kondo physics [1–5] and electron–vibration interaction [6–10]. These junctions are composed of individual molecules suspended between two metallic electrodes. Interestingly, Kondo transport can take place in molecular junctions when an unpaired spin, localized on the molecule, is antiferromagnetically screened by the conduction electrons of the metallic electrodes. This correlated many-body state leads to an enhanced density of states at the Fermi energy, which is expressed as a peak in the conductance at zero voltage (Figure 1a). At a finite voltage and in the absence of inelastic effects, the injected electrons are transmitted off-resonance and the Kondo contribution to the conductance is reduced. However, in the presence of electron–vibration interaction, this picture can change considerably. At a sufficiently high voltage the injected electrons have the necessary energy to excite a certain vibration mode of the molecular junction (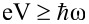). This inelastic process promotes a vibration-mediated Kondo transport for which the excess energy of the injected electrons at finite voltage is released to activate a vibration mode, allowing the inelastic electrons to participate in the Kondo transport. The effect is manifested as enhanced conductance at a finite bias voltage (Figure 1a) corresponding to the relevant vibration energy. The vibration-mediated Kondo effect was mentioned as a possible origin for finite bias peaks that were detected in various molecular junctions [11–13]. This effect provides an attractive way to study electron–vibration interaction in the presence of a many-body electronic system. However, despite many related theoretical studies [14–22], the properties of electron–vibration interaction in the presence of a Kondo process were not addressed extensively by experiments.

Here, we study the interplay between electron–vibration interaction and a Kondo system realized in a molecular junction. Recently, vibration-mediated Kondo transport was demonstrated in junctions consisting of a copper–phthalocyanine (CuPc; Figure 1b) molecule suspended between silver (Ag) electrodes [23]. In the current work we use similar junctions realized in a break junction setup, to analyze the response of vibration-mediated Kondo transport to mechanical stretching. We characterize the evolution of Kondo transport, background conductance and inelastic contributions due to electron–vibration interaction. In particular, the different contributions of Kondo transport and vibration activation can be distinguished. This analysis sheds light on the different parameters that affect vibration-mediated Kondo transport.

**Figure 2:**
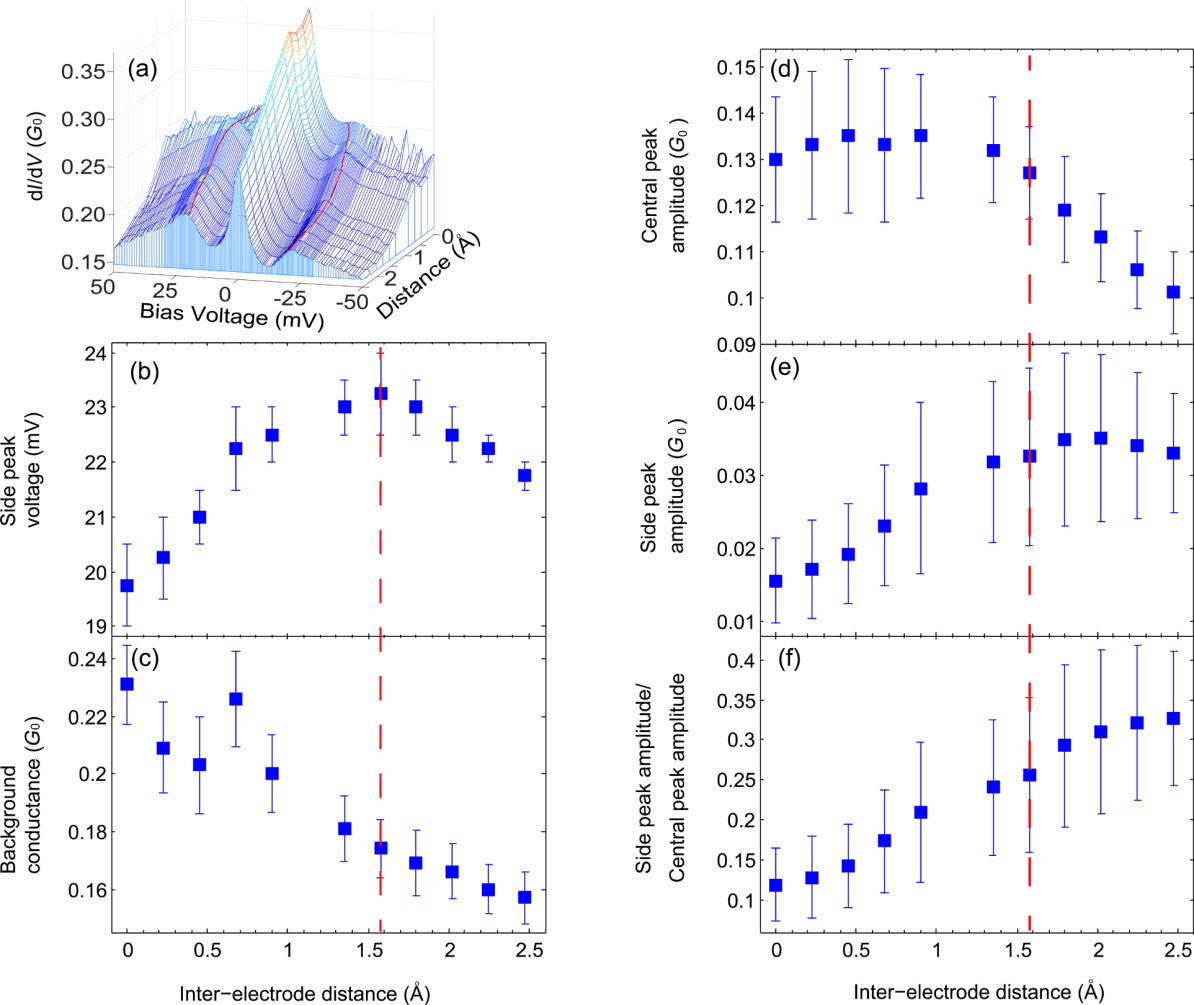
(a) Differential conductance curves measured during the elongation process of the junction. The red lines are a guide to the eye outlining the changing trend of the voltage shift of the side peaks. Average side peak voltage (b), background conductance (c), central peak amplitude (d), side peak amplitude (e), and normalized side peak amplitude (f) as a function of inter-electrode distance. The different parameters were obtained by fitting Lorentzian functions to the zero and finite bias peaks. The background conductance was considered as the conductance at 50 mV, far from the peak features. The stretching dependence was observed in more than 30 junctions, all of which showed a 3–5 mV shift in the position of the finite-bias peaks. The full span of the non-monotonic shift was observed for four junctions.

As a starting point we briefly mention the relevant information found by former analysis of the Ag/CuPc molecular junction [23]. Differential conductance measurements revealed a zero-bias peak accompanied by two side peaks as seen in Figure 1a. The zero-bias peak was associated with a Kondo *S* = 1/2 system based on the peak response to variable temperature and magnetic field. According to calculations [23], in a break junction setup the molecule is attached to the electrodes via its nitrogen atoms with insignificant charge transfer as supported by the experimental fit to Kondo *S* = 1/2. This is in contrast to the CuPc adsorption configuration on a flat Ag substrate in a STM setup which leads to Kondo *S* = 1 [24]. The side peaks were related to a Kondo process as well, on the basis of their amplitude response to temperature, yielding a similar Kondo temperature as extracted for the central peak (*T*_k_ = 25 ± 5 K and 21 ± 5 K, respectively). This was also supported by the observation that the side peaks always appear as satellite peaks to the zero-bias Kondo peak, while they could not be detected in the absence of the zero-bias Kondo peak. Based on the following observations, the side peaks were also related to vibration activation, where the peak voltage is associated with the onset voltage of vibration mode activation [17]. In response to junction elongation the zero-bias peak was quenched, possibly due to a change in the configuration of the molecule in the junction. However, the side peaks evolved into conductance steps (Figure 1a, inset), which are often regarded as the signature of inelastic vibration activation in molecular junctions [6,25–29]. Furthermore, the side peak voltage shifts in a non-monotonic manner in response to moderate elongation (Figure 2a,b). This shift was associated by calculations with the response of a specific junction vibration mode, which is a derivative of the *A*_1_*_g_* gas phase vibration of CuPc, to junction stretching. The process can be intuitively understood in the context of a vibration mode with a transverse component. In analogy to the higher pitch that a guitar string yields in response to stretching, mechanical elongation of the junction leads to a higher vibration frequency and hence to a higher vibration energy. However, further stretching weakens the molecule–electrode binding, resulting in a decrease in vibration energy [26–28].

## Experimental

Ag/CuPc molecular junctions were constructed using a mechanically controllable break junction [30] (Figure 1b) at 4.2 K. A notch was cut in the middle of a Ag wire (99.997%, 0.1 mm diameter, Goodfellow), which was attached to a flexible substrate. A three-point bending configuration under cryogenic vacuum conditions was used in order to break the wire at the notch to form an adjustable gap between two ultra clean Ag tips. A piezoelectric element was used for fine bending of the substrate in order to control the distance between the electrodes with sub-angstrom resolution, in a similar manner as described in [28]. The CuPc molecules (99.95% purity, Sigma Aldrich, further purified in situ), were introduced into the cold junction using a heated local molecular source.

## Results and Discussion

**Figure 3:**
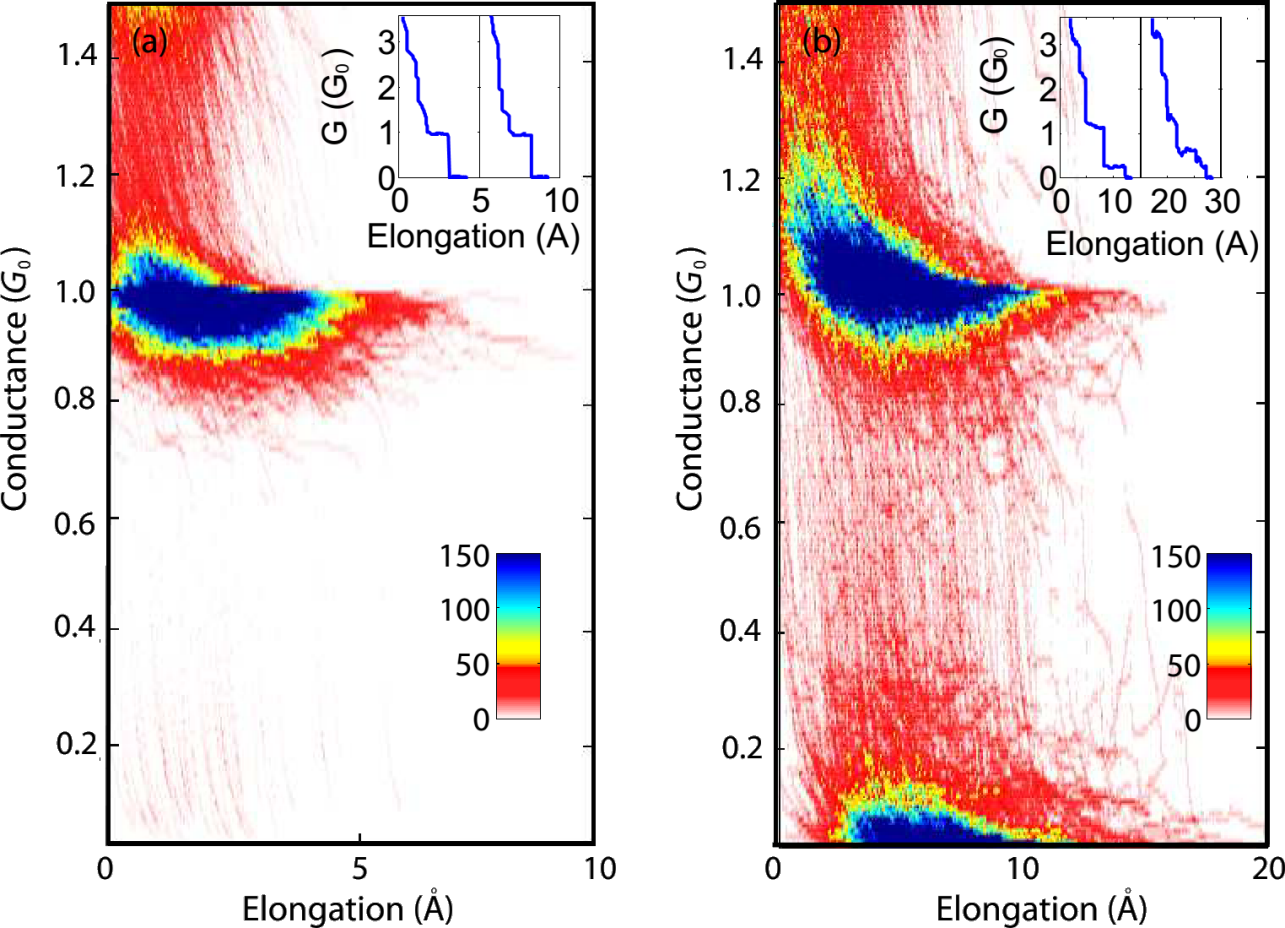
Conductance–length histograms, indicating the most probable conductance of the junction during the final stages of junction elongation before (a) and after (b) CuPc insertion. Both histograms were taken at a voltage of 50 mV, and the lower limit of the conductance scale was chosen as 0.02*G*_0_ to avoid contributions from instrumental noise. Insets: relevant representative conductance traces, measured while elongating the atomic (inset in (a)) and molecular (inset in (b)) junctions. The traces are shifted for clarity by 5 and 15 Å, respectively.

The bare Ag junctions were analyzed before the introduction of CuPc. Figure 3a shows a conductance–length histogram that indicates the most probable conductance of the junction (at a voltage of 50 mV), focusing on the final stages of junction elongation before rupture. A typical conductance of approx. 1*G*_0_ is measured right before the junction breaks. This conductance value is ascribed to Ag atomic junctions [31–32]. Further elongation leads to an exponential conductance decay, which is the signature of tunneling transport that follows the contact rupture. After the introduction of CuPc to the bare Ag junction, new counts at conductance values mostly below 0.2*G*_0_ can be observed, as demonstrated in Figure 3b. This is an indication for the insertion of molecules into the junction. The formation of molecular junctions was further verified by differential conductance measurements as a function of voltage that revealed either Kondo peaks at zero and finite bias (Figure 1a) or vibration-induced conductance steps at the same voltage of the side peaks [23] (Figure 1a, inset) in about 30% of junctions, with a total of 186 junctions exhibiting a Kondo peak accompanied by satellite peaks and 31 junctions exhibiting a vibration-induced conductance step. These peaks and steps were not observed for the bare Ag junctions.

We focus on the evolution of differential conductance curves while gradually increasing the inter-electrode separation in increments of 0.2 Å. After each elongation step, the inter-electrode separation was fixed and a differential conductance spectrum was measured (Figure 2a). The amplitude of the zero and finite-bias peaks were obtained by fitting a Lorentzian on top of a constant background conductance. The latter is determined away from the peak features, at 50 mV. As the junction is stretched, the background conductance is reduced almost linearly (Figure 2c), implying a simple relation between the background non-Kondo transmission and the elongation of the metal–molecule–metal bridge. In contrast, the amplitude of the central Kondo peak (Figure 2d) shows two distinguished trends in response to junction elongation. Along the first 1.6 Å the zero-bias peak is rather insensitive to junction elongation. However, further stretching reduces the peak amplitude. The response of a zero-bias peak to junction elongation was also studied in other molecular junctions [33–34].

Remarkably, the reduction in the central peak amplitude begins at the same inter-electrode distance (dashed red line) in which the side-peak voltage changes its course to a voltage shift towards lower values (Figure 2b). As was mentioned above, the latter is ascribed to metal–molecule bond rupture. This process can also lead to lower Kondo transport by reducing the coupling of the relevant molecular orbitals to at least one of the electrodes [12]. Former ab initio calculations found a good coupling between a certain vibration mode that shows non-monotonic response to stretching and the molecular orbital on which the single spin is localized [23]. Such coupling may explain why two different quantities, namely, the side peak voltage that indicates the onset of vibration activation and the zero-bias Kondo amplitude, change their evolution trends at the same inter-electrode distance. We note that side peaks accompanying a Kondo resonance, that show a monotonous shift in response to junction stretching, were previously reported [12].

The background and the Kondo conductance contributions stem from transport through different molecular orbitals, while direct tunneling between the Ag electrodes can also be considered as a source for background conduction [35]. We speculate that the different response of the background and the Kondo conductance to junction elongation (Figure 2c,d) comes from the different response of the orbital hybridization at the metal–molecule interface to mechanical manipulations.

The evolution of the side peak amplitude as a function of junction elongation is presented in Figure 2e. In contrast to the central peak response, the side peak amplitude increases along the whole elongation process with a transition to a more moderate slope for junction elongation larger than 1.6 Å. Since the origin of the side peaks is related to vibration activation as well as to Kondo transport, both effects contribute to the overall response of the side peaks to junction elongation. To eliminate the Kondo contribution, the side peak amplitude was normalized by the central Kondo peak amplitude at each inter-electrode distance as presented in Figure 2f. Remarkably, the normalized amplitude of the side peaks increases linearly along the whole elongation process. This behavior reflects the response of the inelastic conductance contribution, which stems from electron–vibration interaction, to junction stretching. Interestingly, for off-resonance electron–vibration interaction, it was shown that stretching of simple atomic scale junctions such as suspended gold atomic chains or molecular junctions based on *n*-alkanes can increase the inelastic conductance in a similar way, possibly as a response to variations in the electronic structure of the stretched junction [27,36–38]. The examined evolution of the side peaks due to junction elongation reflects a simple linear response of inelastic conductance due to electron–vibration interaction as well as a more complicated response of the Kondo transport to mechanical stretching.

## Conclusion

We analyzed the transport characteristics of molecular junctions displaying a vibration-mediated Kondo effect. The influence of junction stretching on the Kondo conductance, background conductance and the inelastic contribution due to electron–vibration interaction was revealed and the relation between the different processes was pointed out. We anticipate that our analysis will allow for a better understanding of the vibration-mediated Kondo process and its interplay with the zero-bias Kondo process.
